# Fluorene exposure among PAH-exposed workers is associated with epigenetic markers related to lung cancer

**DOI:** 10.1136/oemed-2020-106413

**Published:** 2020-05-08

**Authors:** Ayman Alhamdow, Yona J Essig, Annette M Krais, Per Gustavsson, Håkan Tinnerberg, Christian H Lindh, Jessika Hagberg, Pål Graff, Maria Albin, Karin Broberg

**Affiliations:** 1 Institute of Environmental Medicine, Karolinska Institutet, Stockholm, Sweden; 2 Department of Laboratory Medicine, Division of Occupational and Environmental Medicine, Lund University, Lund, Sweden; 3 Region Stockholm, Centre for Occupational and Environmental Medicine, Stockholm, Sweden; 4 School of Public Health and Community Medicine, Section of Occupational and Environmental Medicine, University of Gothenburg Sahlgrenska Academy, Göteborg, Sweden; 5 MTM Research Centre, Örebro universitet Akademin för Naturvetenskap och Teknik, Orebro, Sweden; 6 Department of Occupational and Environmental Medicine, Faculty of Medicine and Health, Örebro University, Örebro, Sweden; 7 Department of Chemical and Biological Work Environment, STAMI, Oslo, Norway

**Keywords:** smoking, health care workers, risk assessment, cross sectional studies, cancer

## Abstract

**Objectives:**

Exposure to high-molecular-weight polycyclic aromatic hydrocarbons (PAHs) may cause cancer in chimney sweeps and creosote-exposed workers, however, knowledge about exposure to low-molecular-weight PAHs in relation to cancer risk is limited. In this study, we aimed to investigate occupational exposure to the low-molecular-weight PAHs phenanthrene and fluorene in relation to different cancer biomarkers.

**Methods:**

We recruited 151 chimney sweeps, 19 creosote-exposed workers and 152 unexposed workers (controls), all men. We measured monohydroxylated metabolites of phenanthrene and fluorene in urine using liquid chromatography coupled to tandem mass spectrometry. We measured, in peripheral blood, the cancer biomarkers telomere length and mitochondrial DNA copy number using quantitative PCR; and DNA methylation of *F2RL3* and *AHRR* using pyrosequencing.

**Results:**

Median PAH metabolite concentrations were higher among chimney sweeps (up to 3 times) and creosote-exposed workers (up to 353 times), compared with controls (p<0.001; adjusted for age and smoking). ∑OH-fluorene (sum of 2-hydroxyfluorene and 3-hydroxyfluorene) showed inverse associations with percentage DNA methylation of *F2RL3* and *AHRR* in chimney sweeps (B (95% CI)=–2.7 (–3.9 to –1.5) for *F2RL3*_cg03636183, and –7.1 (–9.6 to –4.7) for *AHRR*_cg05575921: adjusted for age and smoking), but not in creosote-exposed workers. In addition, ∑OH-fluorene showed a 42% mediation effect on the inverse association between being a chimney sweep and DNA methylation of *AHRR* CpG2.

**Conclusions:**

Chimney sweeps and creosote-exposed workers were occupationally exposed to low-molecular-weight PAHs. Increasing fluorene exposure, among chimney sweeps, was associated with lower DNA methylation of *F2RL3* and *AHRR*, markers for increased lung cancer risk. These findings warrant further investigation of fluorene exposure and toxicity.

**Key messages**

**What is already known about this subject?**

- Occupational exposure to high-molecular-weight polycyclic aromatic hydrocarbons (PAHs) has been widely studied in relation to cancer biomarkers, however, less is known about exposure to low-molecular-weight PAHs and their carcinogenicity.
- Workers exposed to PAHs are at a higher risk of cancer.
- Hypomethylation of *F2RL3* and *AHRR* has been shown to be marker for lung cancer risk.

**What are the new findings?**

- Chimney sweeps and creosote-exposed workers were exposed to the low-molecular-weight PAHs fluorene and phenanthrene.
- Fluorene exposure was associated with the hypomethylation of *F2RL3* and *AHRR*.

**How might this impact policy or clinical practice in the foreseeable future?**

- This study highlights the importance of measuring not only high-molecular-weight PAHs but also the low-molecular-weight PAHs when assessing occupational PAH exposure.
- Further, it indicates that even exposure to low-molecular-weight PAHs might have a carcinogenic effect. If this is the case, then the number of workers under risk is much larger than currently assumed, as many workers are exposed to low-molecular-weight PAHs only.

## Introduction

Scrotal cancer among chimney sweeps was the first cancer form linked to chemical exposure.^1^ Even today, the risk of occupational cancer among chimney sweeps is very high, based on analysis of 53 occupational groups (n=15 million individuals) from the Nordic countries (Sweden, Norway, Finland, Denmark and Iceland).^2^ For instance, standardised incidence ratio (SIR) and 95% CI for chimney sweeps in all countries were SIR 2.41 (95% CI: 1.40 to 3.85) for pharynx cancer, and SIR 1.86 (95% CI: 1.19 to 2.76) for oesophageal cancer.^2^ Moreover, high risk for cancer was seen in a cohort of chimney sweeps (n=6320) from Sweden (eg, SIR=1.30 (95% CI: 1.21 to 1.39) for total cancer; and SIR=2.08 (95% CI: 1.19 to 3.38) for oesophageal cancer).^3^ The same study found that the number of working years as a chimney sweep (a proxy for soot exposure) was positively associated with cancer incidence.^3^ The major carcinogenic exposure for the chimney sweeps is considered to be polycyclic aromatic hydrocarbons (PAHs)—the main constituent of soot.^4 5^ The International Agency for Research on Cancer (IARC) has classified PAHs as human carcinogens (group 1: eg, benzo[a]pyrene; BaP), probable human carcinogens (group 2A: eg, dibenz[a, h]anthracene), possible human carcinogens (group 2B: eg, benz[a]anthracene; BaA) or not classifiable as to their carcinogenicity to humans (group 3: eg, pyrene, phenanthrene (Phe) and fluorene (Flu)).^6^ Creosote-impregnating workers is another occupational group exposed to PAHs with an elevated risk for cancer.^6 7^ Workers in this occupation are exposed to PAHs from creosote oil, which is classified as a probable human carcinogen.^6^

We previously demonstrated, by measuring four PAH metabolites in urine, that currently working chimney sweeps and creosote-exposed workers in Sweden were exposed to the PAHs pyrene and phenanthrene, BaA and BaP (BaP was only found in chimney sweeps).^4 8^ Notably, the highest PAH exposure, measured as urinary PAH metabolites, found in creosote-exposed workers was phenanthrene—a low-molecular-weight PAH (median 2-OH-phenanthrene concentrations in urine were 29.6 vs 0.14 µg/g creatinine for creosote-exposed workers and controls, respectively). Phenanthrene and other low-molecular-weight PAHs, such as fluorene, might thus be interesting targets for biomonitoring studies of PAH-exposed workers. We also showed that both chimney sweeps and creosote-exposed workers had lower DNA methylation of the genes *F2RL3* and *AHRR*—prospective biomarkers for lung cancer.^8 9^ We did not find any associations with relative telomere length (TL) or mitochondrial DNA copy number (mtDNAcn), two biomarkers previously linked to cancer risk,^10 11^ nor did we observe associations between the four measured PAH metabolites and DNA methylation levels.^8^ The latter observation may be due to differences in half-lives between the urinary PAH metabolites and DNA methylation, or alternatively, that the four PAHs analysed do not cause epigenetic changes. It therefore remains to clarify if PAHs present in soot and creosote oil can cause the cancer-related DNA methylation. This will increase our knowledge of occupational PAH exposure and related risk of cancer.

We aimed in this study to (i) further characterise the exposure to phenanthrene, (ii) investigate exposure to fluorene and (iii) evaluate the associations of phenanthrene and fluorene exposures with early biomarkers of cancer (TL, mtDNAcn and DNA methylation), among chimney sweeps, creosote-exposed workers and controls.

## Methods

### Study design and participants

This is a cross-sectional study based on the following occupational groups: chimney sweeps (n=151), control workers occupationally unexposed to PAHs (n=152) and a small group of creosote-exposed workers (n=19); all men and working in Sweden. The latter group was included due to their known high exposure to PAHs from the creosote oil, which gives us the possibility to evaluate both moderate (chimney sweeps) and high (creosote-exposed workers) PAH exposure. Detailed information about study groups, including recruitment, work tasks and sampling, have previously been described.^4 8^ In brief, the recruitment process took place during 2010–2015 for chimney sweeps and controls and during 2013–2016 for creosote-exposed workers. The company response rate was 87% for chimney sweeps, 58% for controls and 100% for creosote-exposed workers.^4^ Company visits were performed in the afternoons of Wednesdays or Thursdays for chimney sweeps, throughout the week for controls, and on Thursdays for creosote-exposed workers, during which trained nurses collected questionnaires, sampled blood and urine, and measured weight and height. The questionnaire included questions about age, chronic diseases, level of education, tobacco smoking history, intake of dietary items including vegetables, fruits and fish, use of chewing tobacco (snus), passive smoking, physical activity, residential history, employment history and exposure from hobbies. The questionnaire of chimney sweeps further explored the extent of workers’ engagement in different sweeping tasks (eg, soot sweeping in private houses as well as in industrial buildings) during different time periods (eg, during the past 12 months).

Postshift spot urine and EDTA blood samples were collected from participants and transported at room temperature to the Division of Occupational and Environmental Medicine, Lund University for storage at −20°C. Five chimney sweeps and four controls refrained from donating blood samples, whereas three chimney sweeps and one control could not urinate. All creosote-exposed workers donated blood and urine samples. Written informed consent was obtained from all study participants. The study was carried out in accordance with the ethical guidelines of the Declaration of Helsinki 1964 and its later amendments and was approved by the Regional Ethics Committee in Lund, Sweden and the Regional Ethics Committee in Uppsala, Sweden.

### PAH metabolites in urine

We measured urinary monohydroxylated metabolites of phenanthrene (1-OH-Phe, 2-OH-Phe, 3-OH-Phe and 4-OH-Phe) and fluorene (2-OH-Flu and 3-OH-Flu) by liquid chromatography coupled to tandem mass spectrometry (LC-MS/MS). Details of the method were described previously.^4^ Urine samples (0.2 mL) were pipetted into a 96-well plate and hydrolysed by adding 0.1 mL of ammonium acetate (pH 6.5) and 0.01 mL of β-glucuronidase (*Escherichia coli*). The solution was incubated for 30 min at 37°C. Afterwards, 0.025 mL of a 50:50 (v/v) water/acetonitrile solution and 0.025 mL of deuterium-labelled internal standards (D_9_-1-OH-Phe, D_9_-2-OH-Phe, D_9_-4-OH-Phe, D_9_-2-OH-Flu; Toronto Research Chemicals, North York, Ontario, Canada) were added (final concentrations 5 ng/mL). Plates were centrifuged for 10 min at 3000 rpm, thereafter, 3 µL of each sample were injected onto the column and analysed using a Shimadzu UFLC system (Shimadzu Corporation, Kyoto, Japan), coupled to QTRAP6500+ (AB Sciex, Foster City, California, USA) and equipped with a Turbolon Spray source (AB Sciex). The separation was achieved using a Kinetex C18 column (2.6 µm, 100 Å, 2.1 mm i.d. × 100 mm, Phenomenex, Torrance, California, USA). The mobile phase was water (A) and acetonitrile (B). The gradient was linearly increased from 5% B to 75% B within 6 min, then to 95% B within 10 s and held there for 1 min. 2-OH-Phe and 3-OH-Phe as well as 2-OH-Flu and 3-OH-Flu could not be separated and were therefore analysed as single peaks (∑2-OH-Phe, 3-OH-Phe and ∑OH-Flu, respectively). Samples were prepared and run in duplicates and the average values were used for further analysis. Limit of detection (LOD) values were calculated from water blanks. All PAH metabolite concentrations were adjusted to urinary creatinine values (µg/g crea.).

### DNA methylation of *F2RL3* and *AHRR*

DNA methylation data used in this study were reported in a previous publication.^8^ Hypomethylation of the CpG cg03636183 (Illumina 450K bead chip ID) in exon 2 of *F2RL3* (encoding the coagulation factor II (thrombin) receptor-like 3) and cg05575921 in intron 3 of *AHRR* (encoding the Aryl Hydrocarbon Receptor Repressor) is strongly associated with tobacco smoking and has in prospective studies been associated with increased lung cancer risk.^9 12^ We analysed DNA methylation of two CpGs in *F2RL3* and three CpGs in *AHRR*, including cg03636183 and cg05575921. A detailed description of the method is previously provided.^8^ In brief, DNA samples (500 ng), extracted from peripheral blood, were bisulfite-treated using EZ-96 DNA Methylation-Gold Kit (Zymo Research, Irvine, California, USA). All samples were randomised and DNA methylation controls (0% and 100% methylation; Qiagen GmbH, Hilden, Germany) were added to 96-well plates. Bisulfite-treated DNA sequences of interest were amplified using the thermal cycler (SimpliAmp; Thermo Fisher Scientific, Carlsbad, California, USA) and DNA methylation was measured using PyroMark Q96 ID (Qiagen). All PCR and pyrosequencing chemicals and reagents were purchased from Qiagen except for Streptavidin Sepharose High Performance (GE Healthcare, Uppsala, Sweden). We randomly selected and reran 10% of the samples to calculate the coefficient of variation for each CpG (ranged between 2.5% and 4.1%).

### Relative TL and mtDNAcn

Data of relative TL and relative mtDNAcn were reported previously.^8^ Measurement was performed by quantitative PCR (qPCR) using DNA samples extracted from peripheral blood. The copy number of telomere repeats (*T*), copy number of mitochondrial DNA (*M*) and copy number of a single copy gene (*HBB*: haemoglobin beta) (*S*) were calculated from independent qPCR runs using a standard curve. Thereafter, relative TL was calculated as the ratio T/S and relative mtDNAcn as the ratio *M*/*S*. All study samples, negative controls and standard curve DNA were run in triplicates (SD <0.2 was accepted for *C_t_* values of the triplicates) and 10% of the samples were randomly selected and rerun to ensure quality control. The coefficient of variation was 7.3% for relative TL and 14.4% for relative mtDNAcn. The coefficient of determination (R^2^) for all qPCR runs of the telomere, *HBB* and mtDNAcn was >0.99. Further information about PCR conditions, standard curve and primer sequences is described elsewhere.^8^

### Statistical analysis

Median, minimum, and maximum values were calculated for the continuous variables (age, body mass index (BMI), cancer biomarkers, and PAH metabolites). Frequencies (percentages) were calculated for categorical variables (eg, use of snus and cigarette smoking). Differences between study groups were evaluated by the Kruskal-Wallis test for continuous variables and by the Fisher’s exact test for categorical variables. To evaluate PAH metabolite concentration differences between study groups, age-adjusted and smoking-adjusted linear regression models (analysis of all participants) or age-adjusted models (analysis in each smoking stratum) were used. Intercorrelations of PAH metabolites, including high-molecular-weight metabolites that were analysed in a previous publication,^8^ were evaluated by Spearman’s correlations and presented for former/never smoking chimney sweeps and controls. The sum of all metabolites of phenanthrene (∑OH-Phe=1-OH-Phe + ∑2-OH-Phe,3-OH-Phe+4-OH-Phe: µg/g crea.) was calculated in order to compare with other studies. Differences in urinary PAH metabolite concentrations for chimney sweeps who spent ≥50% of their time doing soot sweeping in the past 12 months versus those who spent <50% (excluding current smokers) were presented as boxplots and evaluated by Mann-Whitney U test.

Unadjusted and multivariable linear regression models were fit to evaluate associations between the PAH metabolite concentrations and cancer biomarkers in study groups: model 1, unadjusted; and model 2, adjusted for age and smoking (a priori confounders). Unstandardised beta estimate (*B*) and 95% CI were presented for each association. To disentangle the effect of smoking from the effect of occupational PAH exposure on the association between ∑OH-Flu and DNA methylation, an additional model was fit (model 3) among smoking chimney sweeps (n=27) and smoking controls (n=25) adjusting for age and pack-years. Creosote-exposed workers were not considered in model 3 due to the limited number of smokers (n=3). We adjusted for multiple comparisons (false discovery rate <0.05) in models 1 and 2. Further adjustment was evaluated for use of snus, physical activity, intake of fish, level of education, current residential area, exposure to smoke from a hobby and passive smoking. In addition, the associations between ∑OH-Flu and cancer biomarkers were evaluated among never smoking chimney sweeps and controls adjusting for age. For all linear regression models, key model assumptions were evaluated.

Finally, we analysed whether PAH metabolites are mediating the observed chimney sweeps’ lower DNA methylation of *AHRR* and *F2RL3* (7), that is, chimney sweeping → PAH exposure (PAH metabolite) → lower DNA methylation. Mediation percentage was calculated based on the formula (mediation % = [(*B*_0_−*B*)/*B*_0_] ×100), where *B*_0_ is the unstandardised estimate of the independent variable ‘study group’ in the base model (eg, DNA methylation=intercept + *B*_0_ × study group + *B*_1_ × age), and *B* is the unstandardised estimate of ‘study group’ after introducing the variable (mediator) PAH metabolite (such as ∑OH-Flu) to the model (DNA methylation=intercept + *B* × study group + *B*_1_ × age + *B*_2_ × PAH metabolite).^13^ We only evaluated mediation for CpGs showing p<0.2 in the base model.

All statistical analyses were performed with SPSS statistical software V.25.0. A *P* value <0.05 was considered for statistical significance.

## Results

Basic characteristics of study groups, as well as levels of cancer biomarkers, are described in table 1. Controls had slightly higher BMI compared with chimney sweeps (BMI data were not available for creosote-exposed workers). The use of snus and current residential area differed between study groups. As reported in our previous publication, TL and mtDNAcn did not differ between study groups.^8^ Hypomethylation of *F2RL3* (CpG1 and CpG2_cg03636183) and *AHRR* (CpG1, CpG2, and CpG3_cg05575921) was observed among chimney sweeps and creosote-exposed workers compared with controls (table 1).^8^

**Table 1:** Basic characteristics of study groups

Covariates	Controls (n=152)*	Chimney sweeps (n=151)*	Creosote-exposed workers (n=19)*	P value†
Age (years)	43 (20–63)	43 (19–66)	32 (22–58)	0.492
BMI (kg/m^2^)	27 (20–45)	26 (19–37)	–	0.019
Relative telomere length	0.57 (0.35–1.0)	0.56 (0.36–0.97)	0.57 (0.33–0.78)	0.836
Relative mitochondrial DNA copy number	0.87 (0.54–1.7)	0.83 (0.51–1.41)	0.95 (0.43–1.34)	0.162
*F2RL3*_CpG1	89.5 (58–100)	88.8 (55–99)	87.2 (65–96)	0.019
*F2RL3*_CpG2 (cg03636183)	76.7 (48–86)	76.6 (45–83)	73.3 (50–78)	0.011
*AHRR*_CpG1	75.0 (25–88)	73.0 (21–90)	74.1 (31–83)	0.087
*AHRR*_CpG2	69.1 (23–80)	66.5 (20–77)	68.7 (31–82)	0.004
*AHRR*_CpG3 (cg05575921)	90.0 (35–100)	88.1 (29–100)	84.9 (37–94)	0.006
Cigarette smoking				
Current smoker	25 (16.4)	27 (17.9)	3 (15.8)	0.915
Party/ former smoker	44 (28.9)	49 (32.5)	6 (31.6)	
Never smoker	83 (54.6)	74 (49.0)	9 (47.4)	
Snus (yes)	29 (19.1)	52 (34.4)	6 (31.6)	0.004
Passive smoking (yes)	24 (15.8)	28 (18.5)	5 (26.3)	0.384
Exposure from hobby (yes)	6 (3.9)	9 (6.0)	2 (10.5)	0.268
Vegetables‡ (≥5 times per week)	88 (57.9)	96 (63.6)	11 (57.9)	0.609
Fruits§ (≥5 times per week)	88 (57.9)	75 (49.7)	10 (52.6)	0.351
Fish¶ (≥1 per week)	70 (46.1)	73 (48.3)	9 (47.4)	0.884
Physical activity**(high)	62 (40.8)	73 (48.3)	7 (36.8)	0.370
Education (higher than high school)	31 (20.4)	24 (15.9)	0 (0)††	0.100
Current residency (big city)	70 (46.1)	52 (34.4)	11 (57.9)††	0.006

Values are median (min–max) for continuous variables or n (%) for categorical variable. The content of this table was previously reported (Alhamdow *et al*, 2018).

*Up to two missing cases for some of the variables.

†*P* value of Kruskal-Wallis test for continuous variables and Fisher’s exact test for categorical variables.

‡Intake of all kinds of vegetables, legumes and root vegetables (fresh, frozen, canned, stewed, juice, soup and so on).

§Intake of all kinds of fruits and berries (fresh, frozen, canned, juice, jam and so on).

¶Intake of all kinds of fish.

**At least 30 min per week of sudorific activities such as running and badminton during leisure time.

††Missing data for four participants.

BMI, body mass index.

Chimney sweeps had up to three times higher median concentrations of all urinary PAH metabolites measured (1-OH-Phe; 0.23 µg/g crea., ∑2-OH-Phe, 3-OH-Phe; 0.37 µg/g crea., 4-OH-Phe; 27 ng/g crea., and ∑OH-Flu; 0.32 µg/g crea.) than the controls (0.14 µg/g crea., 0.11 µg/g crea., 15 ng/g crea. and 0.15 µg/g crea., respectively; p<0.001; all participants, adjusting for age and smoking; table 2). Creosote-exposed workers had very high concentrations of all urinary PAH metabolites, for example, up to 353 times higher median concentrations of ∑OH-Flu (53 µg/g crea.) as compared with the controls (p<0.001; all participants, adjusted for age and smoking). The concentrations of 4-OH-Phe among chimney sweeps and controls were very low (unit ng/g crea.).

**Table 2:** Median concentrations of urinary PAH metabolites for study groups

PAH metabolite	Controls				Chimney sweeps					Creosote-exposed workers				
	*n*	*Median*	*Min*	*Max*	*n* *	*Median*	*Min*	*Max*	*P value* *†*	*n* *	*Median*	*Min*	*Max*	*P value* *‡*
1-OH-Phe (µg/g crea.)														
All participants	151	**0.14**	0.016	2.3	148	**0.23**	0.032	1.7	<0.001*§*	19	**19**	5.1	61	<0.001*§*
Smokers	24	**0.21**	0.074	0.73	27	**0.30**	0.044	0.67	0.052	3	**27**	20	29	0.005
Party/former smokers	44	**0.15**	0.030	0.85	49	**0.28**	0.053	1.7	<0.001	6	**16**	5	29	<0.001
Never smokers	83	**0.12**	0.016	2.3	71	**0.18**	0.032	1.3	<0.001	9	**14**	6	55	<0.001
∑2-,3-OH-Phe (µg/g crea.)														
All participants	151	**0.11**	0.027	2.7	148	**0.37**	0.048	4.6	<0.001*§*	19	**35**	11	92	<0.001*§*
Smokers	24	**0.27**	0.041	1.3	27	**0.50**	0.080	2.1	0.004	3	**49**	41	49	0.005
Party/ former smokers	44	**0.11**	0.044	0.62	49	**0.42**	0.055	4.6	<0.001	6	**24**	11	49	<0.001
Never smokers	83	**0.10**	0.027	2.7	71	**0.31**	0.048	3.1	<0.001	9	**28**	13	92	<0.001
4-OH-Phe (ng/g crea.)														
All participants	151	**15**	2.6	95	148	**27**	3.8	304	<0.001*§*	19	**1051**	293	9780	<0.001*§*
Smokers	24	**40**	7.6	95	27	**46**	6.4	152	0.462	3	**961**	759	1144	0.005
Party/ former smokers	44	**16**	2.8	92	49	**30**	3.8	304	<0.001	6	**1186**	293	7513	<0.001
Never smokers	83	**13**	2.6	58	71	**20**	4.4	175	<0.001	9	**1051**	300	9780	<0.001
∑OH-Phe (µg/g crea.)														
All participants	151	**0.27**	0.070	5.1	148	**0.64**	0.086	6.6	<0.001*§*	19	**51**	19	138	<0.001*§*
Smokers	24	**0.50**	0.13	2.1	27	**0.88**	0.13	2.9	0.010	3	**78**	62	78	0.005
Party/ former smokers	44	**0.28**	0.12	1.1	49	**0.71**	0.12	6.6	<0.001	6	**44**	19	72	<0.001
Never smokers	83	**0.23**	0.070	5.1	71	**0.54**	0.086	4.3	<0.001	9	**42**	19	138	<0.001
∑OH-Flu (µg/g crea.)														
All participants	151	**0.15**	0.042	3.8	148	**0.32**	0.063	4.3	<0.001*§*	19	**53**	27	128	<0.001*§*
Smokers	24	**1.4**	0.21	3.8	27	**1.5**	0.22	3.4	0.473	3	**79**	71	118	0.005
Party/ former smokers	44	**0.15**	0.073	0.82	49	**0.39**	0.066	4.3	<0.001	6	**46**	42	70	<0.001
Never smokers	83	**0.13**	0.042	0.78	71	**0.20**	0.063	1.9	<0.001	9	**38**	27	100	<0.001

*There was one participant with missing smoking status.

†General linear model comparing controls with chimney sweeps adjusted for age.

‡General linear model comparing controls with creosote-exposed workers adjusted for age.

§Further adjusted for smoking.

∑OH-Flu, sum of 2-hydroxyfluorene and 3-hydroxyfluorene; ∑OH-Phe, sum of 1-hydroxyphenanthrene, 2-hydroxyphenanthrene, 3-hydroxyphenanthrene and 4-hydroxyphenanthrene; ∑2-,3-OH-Phe, sum of 2-hydroxyphenanthrene and 3-hydroxyphenanthrene; PAH, polycyclic aromatic hydrocarbon.

There were higher median concentrations of all PAH metabolites among chimney sweeps across smoking categories (smokers, party/former smokers and never smokers), compared with the controls (table 2). Smokers (both chimney sweeps and controls) had increased concentrations of ∑OH-Flu in urine compared with the other two smoking categories. The phenanthrene metabolites showed a similar but the less pronounced pattern with smoking categories (table 2). Low-molecular-weight PAH metabolites showed stronger intercorrelations among former/never smoking chimney sweeps (r_S_=0.72–0.92) compared with former/never smoking controls (r_S_=0.40–0.72) (online supplementary table S1). Further, all measured PAH metabolites showed higher concentrations in chimney sweeps who reported high soot sweeping (≥50% of their working time) during the past 12 months compared with those who performed less soot sweeping (<50%) (online supplementary figure S1; p<0.001, Mann-Whitney U test).

10.1136/oemed-2020-106413.supp1 Supplementary data

Linear regression analyses showed strong inverse associations between ∑OH-Flu and DNA methylation of all five analysed CpGs of *F2RL3* and *AHRR* in chimney sweeps (B (95% CI)= –7.1 (–9.6 to –4.7) for *AHRR*_ cg05575921, model 2, table 3, adjusted for age, smoking and FDR) and controls (B (95% CI)= –8.2 (–12.0 to –4.6), *AHRR*_ cg05575921, model 2, table 3, adjusted for age, smoking and FDR), but not in creosote-exposed workers (B (95% CI)= –0.062 (–0.4 to 0.27), *AHRR*_ cg05575921, model 2, table 3, adjusted for age, smoking and FDR). Further adjustment for use of snus, physical activity, intake of fish, level of education, current residential area, exposure to smoke from a hobby and passive smoking did not significantly influence the estimates. Restricting the analysis to smokers adjusting for age and pack-years, ∑OH-Flu showed inverse associations with DNA methylation of all five analysed CpG sites of *F2RL3* and *AHRR* in chimney sweeps and controls, but significance was reached in chimney sweeps only, and the estimates were about twice as large in chimney sweeps (eg, B (95% CI)= –10.0 (–16.0 to –5.1), *AHRR*_CpG3, model 3, table 3) compared with controls (B (95% CI)= –5.5 (–11.0 to 0.10), *AHRR*_CpG3, model 3, table 3). Analysis among never smoking chimney sweeps and controls showed attenuated effect estimates and there was no clear association between ∑OH-Flu and DNA methylation (online supplementary table S2). Metabolites of phenanthrene did not show associations with DNA methylation (online supplementary table S3).

**Table 3:** Linear regression analyses for the associations between urinary concentrations of ∑OH-fluorene (µg/g creatinine) and cancer biomarkers among chimney sweeps, controls and creosote-exposed workers

	Model 1 (unadjusted)		Model 2 (age-adjusted and smoking-adjusted)		Model 3 (age-adjusted and pack-years-adjusted among smokers)	
	B (95% CI)	P value	B (95% CI)	P value	B (95% CI)	P value
Chimney sweeps	***n=143*** *		n=142*		***n=27†***	
Relative telomere length	−0.013 (−0.036 to 0.0096)	0.25	−0.0046 (−0.031 to 0.022)	0.73	−0.019 (−0.073 to 0.034)	0.46
Relative mtDNAcn	−0.0017 (−0.039 to 0.035)	0.93	0.0081 (−0.038 to 0.054)	0.73	0.028 (−0.097 to 0.15)	0.65
*F2RL3*_CpG1	−6.0 (−7.1 to −4.9)	<0.001‡	−4.0 (−5.3 to −2.7)	<0.001‡	−5.9 (−9.5 to −2.3)	0.003
*F2RL3*_CpG2 (cg03636183)	−5.1 (−6.2 to −4.0)	<0.001‡	−2.7 (−3.9 to −1.5)	<0.001‡	−4.2 (−7.6 to −0.8)	0.019
*AHRR*_CpG1	−10 (−12 to to 8.3)	<0.001‡	−5.9 (−8.2 to −3.5)	<0.001‡	−8.2 (−13 to −3.5)	0.002
*AHRR*_CpG2	−10 (−12 to to 8.5)	<0.001‡	−5.8 (−7.8 to −3.8)	<0.001‡	−7.7 (−12 to −3.7)	0.001
*AHRR*_CpG3 (cg05575921)	−14 (−16 to to 12)	<0.001‡	−7.1 (−9.6 to −4.7)	<0.001‡	−10 (−16 to to 5.1)	0.001
Controls	**n=*147*** *		**n=*147*** *		**n=*25***†	
Relative telomere length	−0.016 (−0.044 to 0.012)	0.27	0.049 (−0.0014 to 0.099)	0.06	0.060 (−0.0023 to 0.12)	0.058
Relative mtDNAcn	−0.069 (−0.12 to -0.021)	0.005‡	−0.0038 (−0.091 to 0.084)	0.93	−0.012 (−0.10 to 0.079)	0.78
*F2RL3*_CpG1	−6.5 (−7.8 to −5.1)	<0.001‡	−3.9 (−6.3 to −1.6)	0.001‡	−2.4 (−6.7 to 1.9)	0.26
*F2RL3*_CpG2 (cg03636183)	−6.8 (−8.0 to −5.6)	<0.001‡	−3.7 (−5.7 to −1.8)	<0.001‡	−2.6 (−6.3 to 1.1)	0.15
*AHRR*_CpG1	−12 (−14 to to 9.9)	<0.001‡	−6.8 (−11 to −2.7)	0.001‡	−3.0 (−9.2 to 3.2)	0.32
*AHRR*_CpG2	−11 (−13 to to 8.9)	<0.001‡	−5.7 (-9.3 to -2.0)	0.003‡	−3.3 (−8.9 to 2.3)	0.23
*AHRR*_CpG3 (cg05575921)	−18 (−20 to to 16)	<0.001‡	−8.2 (−12 to −4.6)	<0.001‡	−5.5 (−11 to 0.1)	0.055
Creosote-exposed workers	***n=19***		***n=18***		***n=3***	
Relative telomere length	−0.00045 (−0.0026 to 0.0017)	0.66	−0.001 (−0.0039 to 0.0018)	0.45	–	–
Relative mtDNAcn	0.00088 (−0.0038 to 0.0055)	0.69	−0.00012 (−0.0066 to 0.0063)	0.97	–	–
*F2RL3*_CpG1	−0.10 (−0.24 to 0.037)	0.14	−0.10 (−0.29 to 0.087)	0.27	–	–
*F2RL3*_CpG2 (cg03636183)	−0.086 (−0.22 to 0.045)	0.18	−0.12 (−0.29 to 0.052)	0.16	–	–
*AHRR*_CpG1	−0.081 (−0.36 to 0.20)	0.55	−0.12 (−0.47 to 0.23)	0.47	–	–
*AHRR*_CpG2	−0.079 (−0.35 to 0.19)	0.55	−0.12 (−0.46 to 0.22)	0.46	–	–
*AHRR*_CpG3 (cg05575921)	−0.11 (−0.42 to 0.19)	0.45	−0.062 (−0.40 to 0.27)	0.69	–	–

Model 1: (cancer biomarker: outcome)=intercept + **B**.(∑OH-fluorene).

Model 2: (cancer biomarker: outcome)=intercept + **B**(∑OH-fluorene)+B1.age+B2.smoking.

Model 3: (cancer biomarker: outcome)=intercept + **B**(∑OH-fluorene)+B1.age+B2.(pack-years).

*There was one missing case for some of the outcomes (eg, *AHRR*_CpG3_cg05575921).

†There were two missing cases among chimney sweeps and four missing cases among the controls.

‡Significant after adjustment for multiple comparisons (FDR<0.05)

B, unstandardised beta estimate; mtDNAcn, mitochondrial DNA copy number; ∑OH-fluorene, sum of 2-hydroxyfluorene and 3-hydroxyfluorene.

In the mediation analysis, the hypomethylation of *AHRR* CpG2 was significantly associated with being a chimney sweep in the base model, and the effect estimate (ie, B_0_) was attenuated by 42% when ∑OH-Flu was added to the model (table 4). A marked attenuation was also observed for the other CpGs, and the *P* values of the effect estimates were considerably larger after adding ∑OH-Flu to the model. For example, B estimate (95% CI) for *AHRR*_cg05575921 changed from –2.0 (–4.2 to 0.063) to –0.64 (–2.6 to 1.3) (table 4).

**Table 4:** Mediation analysis for the path (being a chimney sweeps → PAH exposure → DNA hypomethylation), where being a chimney sweep is a binary variable (chimney sweep vs control), PAH exposure is a continuous variable (PAH metabolites) and DNA hypomethylation is a continuous variable (methylation of *F2RL3* and *AHRR*)

Base model†	*F2RL3*_CpG1		*F2RL3*_cg03636183*		*AHRR*_CpG1		*AHRR*_CpG2		*AHRR_*cg05575921	
	B (95% CI)	Mediation%	B (95% CI)	Mediation%	B (95% CI)	Mediation%	B (95% CI)	Mediation%	B (95% CI)	Mediation%
	−0.98 (−2.2 to 0.22)		0.02 (−1.02 to 1.07)	–	−1.5 (−3.6 to 0.54)		−2.6 (−4.4 to −0.69)		−2.0 (−4.2 to 0.063)	
1-OH-Phe (µg/g crea.)‡	−0.91 (−2.2 to 0.34)	7	–	–	−1.7 (−3.8 to 0.49)	-8	−2.7 (−4.6 to −0.76)	-6	−2.2 (−4.3 to 0.038)	-6
∑2-,3-OH-Phe (µg/g crea‡	−0.73 (−2.0 to 0.56)	26	–	–	−1.7 (−4.0 to 0.52)	−12	−2.7 (−4.7 to −0.72)	-7	−2.1 (−4.4 to 0.18)	-3
4-OH-Phe (ng/g crea.)‡	−0.79 (−2.0 to 0.46)	20	–	–	−1.7 (−3.8 to 0.49)	-8	−2.6 (−4.5 to −0.64)	-1	−2.0 (−4.2 to 0.19)	1
∑OH-Phe (µg/g crea.)‡	−0.77 (−2.05 to 0.51)	21	–	–	−1.7 (−3.9 to 0.52)	−10	−2.7 (−4.7 to −0.72)	-6	−2.1 (−4.4 to 0.15)	-3
∑OH-Flu (µg/g crea.)‡	−0.30 (−1.4 to 0.84)	69	–	–	−0.40 (−2.4 to 1.6)	74	−1.5 (−3.3 to 0.31)	42	−0.64 (−2.6 to 1.3)	69

*Mediation analysis was not performed for *F2RL3*_cg03636183 because there was no association with exposure group (p=0.96).

†Outcome (DNA methylation)=intercept + **B**.exposure group (chimney sweep vs control)+B1.age+B2.smoking (three categories).

‡Outcome (DNA methylation)=intercept + **B**.exposure group (chimney sweep vs control)+B1.age+B2.smoking (three categories)+B3.(PAH metabolite).

B, unstandardised beta estimate; ∑OH-Flu, sum of 2-hydroxyfluorene and 3-hydroxyfluorene; ∑OH-Phe, sum of 1-hydroxyphenanthrene, 2-hydroxyphenanthrene, 3-hydroxyphenanthrene, and 4-hydroxyphenanthrene; ∑2-,3-OH-Phe, sum of 2-hydroxyphenanthrene and 3-hydroxyphenanthrene.

## Discussion

In this study, we showed that chimney sweeps and particularly creosote-exposed workers are occupationally exposed to the low-molecular-weight PAHs phenanthrene and fluorene. Moreover, chimney sweeps showed the hypomethylation of *F2RL3* and *AHRR*—prospective markers for increased risk of lung cancer,^9^ with increasing fluorene metabolite concentrations. These results remained after sensitivity analyses among currently smoking chimney sweeps and controls, where potential residual confounding of pack-years of smoking could be taken into account. The association between fluorene and DNA methylation seems to be fluorene-specific as other PAH metabolites, analysed here (metabolites of phenanthrene) or in our previous study (pyrene, BaP and BaA),^8^ did not show such association.

Occupational exposure to phenanthrene and fluorene has been given less attention compared with PAHs such as pyrene (widely used as a surrogate for total PAH exposure) and BaP (group-1 carcinogen).^6 14^ A study among firefighters showed high levels of phenanthrene (median sum of urinary 1-OH-Phe, 2-OH-Phe and 3-OH-Phe was up to 3.1 µg/g crea.) and fluorene (∑OH-fluorene up to 0.81 µg/g crea.).^15^ High phenanthrene exposure was also observed among coke oven workers (median sum of urinary 1-OH-Phe, 2-OH-Phe, 3-OH-Phe, 4-OH-Phe and 9-OH-Phe=10.7 µg/g crea.), workers in the production of graphite electrodes (5.8 µg/g crea.) and refractory materials (16.8 µg/g crea.).^16^ Among workers in electrode production, mean urinary concentrations of phenanthrene and fluorene metabolites were two to three times higher in postshift compared with preshift samples.^17^ Further, workers in the production of fireproof stone were exposed to phenanthrene (median 1-OH-Phe, 2-OH-Phe and 4-OH-Phe were 1.9, 1.6 and 0.3 µg/g crea., respectively).^18^ In our study, chimney sweeps who were soot sweeping ≥50% of their working time had significantly higher phenanthrene and fluorene metabolites in their urine compared with chimney sweeps who spent <50% of their working time sweeping soot (more than twofold increase). In addition, comparing the exposure levels in our study with those of the aforementioned occupational groups, it is apparent that the exposure levels of both phenanthrene and fluorene in creosote-exposed workers (median ∑2-OH-Phe, 3-OH-Phe 35 µg/g crea., ∑OH-Flu 53 µg/g crea.), but not in chimney sweeps (median ∑2-OH-Phe, 3-OH-Phe 0.37 µg/g crea., ∑OH-Flu 0.32 µg/g crea.), were higher than all other occupational groups reported.

Another source of phenanthrene and fluorene exposures is cigarette smoking,^19^ which is probably the major source of fluorene exposure among the controls. The mean urinary concentrations of ∑OH-Phe and ∑OH-Flu in a study among daily smokers who smoked ≥5 cigs/day were 2.46 and 1.7 µg/g crea., respectively.^20^ In another study, comparable exposure to fluorene (mean 1.5 µg/L 2-OH-Flu), but not phenanthrene (mean 0.56 µg/L ∑OH-Phe), was reported among daily smokers who smoked ≥5 cigs/day.^21^ The levels of phenanthrene and fluorene exposure in the latter study are consistent with those observed in our study for the smoking controls (median ∑OH-Flu 1.4 µg/g crea.; ∑OH-Phe 0.5 µg/g crea.). However, even the non-smoking controls showed fluorene metabolite concentrations in urine, indicating that there are other sources of fluorene than smoking in the general environment. It should be noted that no significant difference was observed for the urinary concentrations of ∑OH-Flu when comparing smoking controls to smoking chimney sweeps (1.4 vs 1.5 µg/g crea.). These figures show that cigarette smoking has a greater impact on ∑OH-Flu levels than chimney sweeping. Therefore, the question arose whether the association between fluorene and DNA hypomethylation is due to cigarette smoking only or does fluorene occupational exposure play a role?

We evaluated this question in different ways. We started by adjusting for age and smoking in the linear regression models; however, this adjustment did not seem to fully eradicate the effect of smoking on the association between fluorene exposure and DNA methylation, as seen in the models for the controls (table 3; model 2). This may be caused by the residual confounding of smoking, which in this case can be due to differences in pack-years in smokers and former smokers. We, therefore, restricted the analysis to smoking chimney sweeps and smoking controls adjusting for age and pack-years. We found that the negative effect estimates of fluorene metabolite concentrations on DNA methylation were twice as pronounced among smoking chimney sweeps compared with the smoking controls and significance was reached only for chimney sweeps. This indicates that the relationship between fluorene and DNA methylation is not only related to smoking but also to chimney sweeps’ exposure from work. We could not adjust for pack-years of former smokers because we did not have these data for the controls; however, pack-years of former smokers should not be correlated with urinary fluorene metabolite concentrations, a marker for recent fluorene exposure. It is worth mentioning that our analysis would have benefited from adjustment for blood cell composition, however, we do not expect systematic variations in blood cell composition between study groups because of the similarities in age, health status (healthy workers) and smoking status. The inverse association between fluorene and DNA methylation of *F2RL3* and *AHRR* was corroborated by the mediation analyses where fluorene metabolite concentrations showed mediation effect for the associations between chimney sweeping (being a chimney sweep) versus DNA hypomethylation. Nevertheless, these findings should still be cautiously interpreted, as in the analysis of never smoking chimney sweeps and controls, only *AHRR* CpG2 showed significantly lower methylation with increasing ∑OH-Flu in the sweeps.

Even though creosote-exposed workers were highly exposed to fluorene, they did not show any association between fluorene exposure and DNA methylation. Likely, the small sample size limited our possibilities to detect small to moderate effects. But there may also be other possibilities for the lack of associations. Fluorene might be a proxy for other toxicant(s) present in the work environment of the chimney sweeps (eg, black carbon particles), but not in the work environment of the creosote-exposed workers. In both occupations, exposure to PAHs occurs through inhalation and dermal contact, but the predominant route of exposure to certain PAH might differ.^5 22–24^ Inhalation of particles could lead to inflammatory responses that may cause the hypomethylation of the CpG cites examined in this study by yet unknown mechanisms. Indeed, exposure to diesel exhaust has been linked to DNA alterations of CpG sites related to genes involved in inflammation and oxidative stress response.^25^ Chimney sweeps that are mainly exposed to PAHs from soot particles through inhalation could, therefore, show associations between DNA methylation levels and fluorene exposure. Creosote-exposed workers, on the other hand, are predominantly dermally exposed to non-particulate PAHs from creosote oil^5 24^, and thus no such association could be found. In addition, the PAH content varies between soot and creosote oil. For instance, BaP metabolites were found in chimney sweeps’ urine, but not in creosote-exposed workers’ urine.^8^ Still, BaP did not show any association with DNA methylation in our study. It is also possible that there is no relationship between fluorene and DNA methylation at such extreme levels of fluorene exposure among creosote-exposed workers or that the association is non-linear above a certain level of exposure. A study on exposure from cigarette smoking including current smokers (n=172) found that participants in the second quartiles Q2 (27 cigs/day), Q3 (40 cigs/day) and Q4 (53 cigs/day) had comparable hypomethylation of *AHRR* cg05575921 compared with Q1 (19 cigs/day).^26^ Likewise, when examining the cumulative exposure (pack-years), the authors found that participants in Q2 (23 pack-years), Q3 (38 pack-years) and Q4 (57 pack-years) had similar *AHRR* cg05575921 methylation, and concluded that exposure from current smoking impacts DNA methylation, however, very high levels of smoking exposure adds only a negligible effect.^26^

Despite the frequent presence of fluorene in PAH mixtures, only a limited number of studies have explored its toxicity. An in vitro study using Chinese hamster lung cell line reported a clastogenic effect of fluorene only in the presence of metabolic activation (rat S9 mix).^27^ Fluorene also showed axial development toxicity in zebrafish embryos and sea urchin embryos through dysregulation of the β-catenin/Wnt signalling pathway.^28 29^ An in ovo study assessing histopathological changes in the chicken fetal liver on exposure to various compounds found no genotoxicity, measured as DNA adducts, of fluorene.^30 31^ Due to the scarcity of toxicological studies, and thus, the inadequate evidence of fluorene carcinogenicity, IARC has classified fluorene in group 3.^6^ It is biologically plausible that PAHs could induce alterations in DNA methylation. The key metabolic pathway for PAHs is the oxidisation to reactive metabolites (eg, epoxides: phase I) followed by conjugation (eg, glutathione: phase II).^32^ As a result of the phase II metabolism, PAH exposure could lead to elevated levels of glutathione whose biological synthesis is dependent on homocysteine.^33^ This process may result in reduced levels of S-adenosylmethionine (SAM), a key methyl group donor for DNA methylation, and subsequently lead to DNA hypomethylation.^33^

## Conclusions

Chimney sweeps and creosote-exposed workers are occupationally exposed to phenanthrene and fluorene. We report for the first time that fluorene exposure is associated with the DNA hypomethylation of *F2RL3* and *AHRR*, prospective markers for lung cancer, in the chimney sweeps. Further studies are needed to clarify whether fluorene or other PAHs/toxicants are underlying the epigenetic alterations observed in workers exposed to PAHs.
