# Supplementary material for: Fluorene exposure among PAH-exposed workers is associated with epigenetic markers related to lung cancer
Source: Occup Environ Med. 2020 May 8;77(7):488–95. doi: 10.1136/oemed-2020-106413 (PMC7306866; doi:10.1136/oemed-2020-106413)
Supplement: Supplementary data [file oemed-2020-106413supp001.pdf]

Supplementary material

Fluorene exposure among PAH-exposed workers is associated with epigenetic markers related to lung cancer

Ayman Alhamdow[1,†], Yona J. Essig[2,†], Annette M. Krais[2], Per Gustavsson[1,3], Håkan Tinnerberg[4], Christian Lindh[2], Jessika Hagberg[5,6], Pål Graff[7], Maria Albin[1,2,3], Karin Broberg[1,2,*]

1 Institute of Environmental Medicine, Karolinska Institutet, Stockholm, Sweden.
2 Division of Occupational and Environmental Medicine, Department of Laboratory Medicine, Lund University, Lund, Sweden.
3 Centre for Occupational and Environmental Medicine (CAMM), Region Stockholm, Stockholm, Sweden.
4 Section of Occupational and Environmental Medicine, Sahlgrenska Academy at University of Gothenburg, Gothenburg, Sweden.
5 MTM Research Centre, School of Science and Technology, Örebro University, Örebro, Sweden.
6 Department of Occupational and Environmental Medicine, Faculty of Medicine and Health, Örebro University, Örebro, Sweden.
7 National Institute of Occupational Health (STAMI), Oslo, Norway.

[†]These authors contributed equally to this work.

*To whom correspondence should be addressed:
Karin Broberg
Unit of Metals & Health
Institute of Environmental Medicine
Karolinska Institutet
Nobels Väg 13
SE-171 77 Stockholm, Sweden
Tel: +46 8 524 874 05
Fax: +46 8 33 69 81
Email: karin.broberg@ki.se

Alhamdow A, *et al. Occup Environ Med* 2020; 77:488–495. doi: 10.1136/oemed-2020-106413

Table S1. Intercorrelations between PAH metabolites (including high-molecular-weight metabolites measured in a previous study [Alhamdow at al. 2018]) evaluated by Spearman's correlations among former/never smoking chimney sweeps, controls, and creosote-exposed workers.

**Group**			1-OH-Pyr	2-OH-Phe	3-OH-BaP	3-OH-BaA	1-OH-Phe	∑2-,3-OH-Phe	4-OH-Phe	∑OH-Phe
Controls	2-OH-Phe	r_S	0.444							
		P value (2-tailed)	<0.001							
		n	127							
	3-OH-BaP	r_S	-0.025	-0.038						
		P value (2-tailed)	0.800	0.696						
		n	108	108						
	3-OH-BaA	r_S	0.052	0.187	0.380					
		P value (2-tailed)	0.572	0.040	<0.001					
		n	121	121	108					
	1-OH-Phe	r_S	0.388	0.714	-0.038	0.101				
		P value (2-tailed)	<0.001	<0.001	0.695	0.270				
		n	127	127	108	121				
	∑2-,3-OH-Phe	r_S	0.444	0.957	0.017	0.206	0.721			
		P value (2-tailed)	<0.001	<0.001	0.863	0.023	<0.001			
		n	127	127	108	121	127			
	4-OH-Phe	r_S	0.138	0.497	0.033	0.251	0.404	0.538		
		P value (2-tailed)	0.122	<0.001	0.733	0.005	<0.001	<0.001		
		n	127	127	108	121	127	127		
	∑OH-Phe	r_S	0.425	0.866	-0.017	0.177	0.944	0.890	0.541	
		P value (2-tailed)	<0.001	<0.001	0.865	0.052	<0.001	<0.001	<0.001	
		n	127	127	108	121	127	127	127	
	∑OH-Flu	r_S	0.324	0.658	-0.082	0.126	0.622	0.661	0.491	0.675
		P value (2-tailed)	<0.001	<0.001	0.401	0.169	<0.001	<0.001	<0.001	<0.001
		n	127	127	108	121	127	127	127	127
Chimney sweeps	2-OH-Phe	r_S	0.859							
		P value (2-tailed)	<0.001							
		n	120							
	3-OH-BaP	r_S	0.769	0.678						
		P value (2-tailed)	<0.001	<0.001						
		n	111	111						
	3-OH-BaA	r_S	0.853	0.779	0.789					
		P value (2-tailed)	<0.001	<0.001	<0.001					

Alhamdow A, *et al. Occup Environ Med* 2020; 77:488–495. doi: 10.1136/oemed-2020-106413

		n	115	115	111					
	1-OH-Phe	r_S	0.828	0.905	0.614	0.733				
		P value (2-tailed)	<0.001	<0.001	<0.001	<0.001				
		n	120	120	111	115				
	∑2-,3-OH-Phe	r_S	0.888	0.969	0.698	0.782	0.921			
		P value (2-tailed)	<0.001	<0.001	<0.001	<0.001	<0.001			
		n	120	120	111	115	120			
	4-OH-Phe	r_S	0.722	0.872	0.491	0.670	0.881	0.882		
		P value (2-tailed)	<0.001	<0.001	<0.001	<0.001	<0.001	<0.001		
		n	120	120	111	115	120	120		
	∑OH-Phe	r_S	0.874	0.961	0.668	0.774	0.969	0.986	0.908	
		P value (2-tailed)	<0.001	<0.001	<0.001	<0.001	<0.001	<0.001	<0.001	
		n	120	120	111	115	120	120	120	
	∑OH-Flu	r_S	0.702	0.788	0.588	0.661	0.810	0.828	0.723	0.833
		P value (2-tailed)	<0.001	<0.001	<0.001	<0.001	<0.001	<0.001	<0.001	<0.001
		n	120	120	111	115	120	120	120	120
Creosote-exposed workers	2-OH-Phe	r_S	0.707							
		P value (2-tailed)	0.003							
		n	15							
	3-OH-BaP	r_S	-0.275	-0.200						
		P value (2-tailed)	0.321	0.475						
		n	15	15						
	3-OH-BaA	r_S	0.479	0.418	-0.350					
		P value (2-tailed)	0.071	0.121	0.201					
		n	15	15	15					
	1-OH-Phe	r_S	0.532	0.554	-0.550	0.368				
		P value (2-tailed)	0.041	0.032	0.034	0.177				
		n	15	15	15	15				
	∑2-,3-OH-Phe	r_S	0.725	0.971	-0.196	0.421	0.546			
		P value (2-tailed)	0.002	<0.001	0.483	0.118	0.035			
		n	15	15	15	15	15			
	4-OH-Phe	r_S	0.482	0.754	-0.293	0.307	0.718	0.789		
		P value (2-tailed)	0.069	0.001	0.289	0.265	0.003	<0.001		
		n	15	15	15	15	15	15		
	∑OH-Phe	r_S	0.686	0.861	-0.400	0.400	0.868	0.871	0.882	
		P value (2-tailed)	0.005	<0.001	0.140	0.140	<0.001	<0.001	<0.001	

Alhamdow A, *et al. Occup Environ Med* 2020; 77:488–495. doi: 10.1136/oemed-2020-106413

		n	15	15	15	15	15	15	15	
	∑OH-Flu	r_S	0.718	0.650	-0.250	0.411	0.643	0.586	0.468	0.682
		P value (2-tailed)	0.003	0.009	0.369	0.128	0.010	0.022	0.079	0.005
		n	15	15	15	15	15	15	15	15

Abbreviations: 1-OH-Pyr (1-hydroxypyrene), 2-OH-Phe (2- hydroxyphenanthrene), 3-OH-BaP (3-hydroxybenzo[a]pyrene), 3-OH-BaA (3-hydroxybenzo[a]anthracene), ∑2-,3-OH-Phe (sum of 2- and 3-hydroxyphenanthrene), 4-OH-Phe (4-hydroxyphenanthrene), ∑OH-Phe (Sum of 1-, 2-, 3-, and 4-hydroxyphenanthrene), ∑OH-Flu (sum of 2- and 3-hydroxyfluorene).

Alhamdow A, *et al. Occup Environ Med* 2020; 77:488–495. doi: 10.1136/oemed-2020-106413

Table S2. Linear regression analyses for the associations between urinary concentrations of ∑OH-fluorene (sum of 2- and 3-OH fluorene: µg/g creatinine) and cancer biomarkers among never smoking chimney sweeps and controls adjusting for age [B=unstandardised beta estimate, 95%CI= 95% confidence interval].

	Chimney sweeps (n=70)		***Controls (n=83)***	
	B (95% CI) [a]	*P*	B (95% CI) [a]	*P*
Relative telomere length	0.0066 (-0.076, 0.090)	0.87	0.0073 (-0.20, 0.21)	0.94
Relative mtDNAcn	0.021 (-0.094, 0.14)	0.72	-0.046 (-0.35, 0.26)	0.77
*F2RL3*_CpG1	-0.75 (-3.4, 1.9)	0.58	-4.0 (-11, 3.0)	0.26
*F2RL3*_CpG2 (cg03636183)	0.43 (-1.9, 2.7)	0.71	-1.7 (-7.0, 3.7)	0.54
*AHRR*_CpG1	-1.8 (-6.4, 2.7)	0.42	-1.6 (-13, 10.1)	0.78
*AHRR*_CpG2	-4.1 (-8.0, -0.16)	0.042	4.8 (-4.6, 14.3)	0.31
*AHRR*_CpG3 (cg05575921)	-4.3 (-9.7, 1.1)	0.11	-1.4 (-11, 8.1)	0.77

[a]Outcome (DNA methylation) = intercept + **B.**(∑OH-fluorene) + B1.(age)

Alhamdow A, *et al. Occup Environ Med* 2020; 77:488–495. doi: 10.1136/oemed-2020-106413

Table S3. Linear regression analyses for the association between urinary concentrations of PAH metabolites and cancer biomarkers among chimney sweeps and creosote-exposed workers [B=unstandardised beta estimate, 95%CI= 95% confidence interval].

	**Model 1 (unadjusted)**		**Model 2 (age- and smoking-adjusted)**	
	B (95% CI)	*P*	B (95% CI)	*P*
Chimney sweeps	***n*=143***		***n*=142***	
**1-OH-Phe (µg/g crea.)**				
Relative telomere length	0.0090 (-0.055, 0.072)	0.79	0.022 (-0.038, 0.081)	0.47
Relative mtDNAcn	-0.013 (-0.12, 0.091)	0.81	-0.0048 (-0.11, 0.10)	0.93
*F2RL3*_CpG1	-1.9 (-6.1, 2.2)	0.36	-1.6 (-4.9, 1.7)	0.33
*F2RL3*_CpG2 (cg03636183)	0.090 (-3.7, 3.9)	0.96	0.17 (-2.7, 3.1)	0.91
*AHRR*_CpG1	1.9 (-5.6, 9.4)	0.61	2.7 (-3.0, 8.3)	0.36
*AHRR*_CpG2	0.94 (-6.2, 8.0)	0.79	1.9 (-3.2, 6.9)	0.46
*AHRR*_CpG3 (cg05575921)	0.60 (-8.8, 10)	0.90	1.9 (-4.3, 8.0)	0.55
**∑2-,3-OH-Phe (µg/g crea.)**				
Relative telomere length	0.0070 (-0.022, 0.035)	0.65	0.013 (-0.013, 0.039)	0.33
Relative mtDNAcn	-0.0092 (-0.056, 0.037)	0.70	-0.0042 (-0.051, 0.042)	0.86
*F2RL3*_CpG1	-1.2 (-3.1, 0.67)	0.21	-0.96 (-2.4, 0.50)	0.20
*F2RL3*_CpG2 (cg03636183)	-0.17 (-1.9, 1.6)	0.85	-0.06 (-1.3, 1.2)	0.93
*AHRR*_CpG1	0.66 (-2.7, 4.0)	0.70	1.1 (-1.4, 3.6)	0.39
*AHRR*_CpG2	0.33 (-2.9, 3.5)	0.84	0.77 (-1.5, 3.0)	0.50
*AHRR*_CpG3 (cg05575921)	-0.041 (-4.3, 4.2)	0.98	0.58 (-2.2, 3.3)	0.67
**4-OH-Phe (ng/g crea.)**				
Relative telomere length	0.000079 (-0.00036, 0.00052)	0.72	0.00021 (-0.00020, 0.00062)	0.32
Relative mtDNAcn	0.00020 (-0.00051, 0.00090)	0.58	0.00029 (-0.00042, 0.0010)	0.43
*F2RL3*_CpG1	-0.022 (-0.050, 0.0068)	0.14	-0.011 (-0.034, 0.011)	0.32
*F2RL3*_CpG2 (cg03636183)	-0.011 (-0.038, 0.015)	0.40	-0.0040 (-0.024, 0.016)	0.69
*AHRR*_CpG1	-0.0090 (-0.061, 0.043)	0.73	0.010 (-0.029, 0.049)	0.61
*AHRR*_CpG2	-0.014 (-0.063, 0.035)	0.56	0.0051 (-0.030, 0.040)	0.77
*AHRR*_CpG3 (cg05575921)	-0.022 (-0.087, 0.042)	0.49	0.0053 (-0.037, 0.048)	0.81
**∑OH-Phe (µg/g crea.)**				
Relative telomere length	0.0040 (-0.015, 0.023)	0.69	0.0084 (-0.0096, 0.026)	0.36
Relative mtDNAcn	-0.0050 (-0.036, 0.026)	0.75	-0.0018 (-0.034, 0.030)	0.91
*F2RL3*_CpG1	-0.77 (-2.0, 0.49)	0.23	-0.61 (-1.6, 0.38)	0.23
*F2RL3*_CpG2 (cg03636183)	-0.089 (-1.3, 1.1)	0.88	-0.020 (-0.89, 0.85)	0.96
*AHRR*_CpG1	0.46 (-1.8, 2.8)	0.69	0.77 (-0.95, 2.5)	0.38
*AHRR*_CpG2	0.21 (-2.0, 2.4)	0.85	0.54 (-1.0, 2.1)	0.49
*AHRR*_CpG3 (cg05575921)	-0.0068 (-2.9, 2.8)	1.0	0.45 (-1.4, 2.3)	0.63
Creosote-exposed workers	***n*=19**		***n*=18**	

Alhamdow A, *et al. Occup Environ Med* 2020; 77:488–495. doi: 10.1136/oemed-2020-106413

**1-OH-Phe (µg/g crea.)**				
Relative telomere length	0.00053 (-0.0034, 0.0045)	0.78	-0.000041 (-0.0049, 0.0049)	0.99
Relative mtDNAcn	0.0026 (-0.0059, 0.011)	0.53	0.0019 (-0.0085, 0.012)	0.70
*F2RL3*_CpG1	-0.036 (-0.31, 0.24)	0.78	-0.07 (-0.40, 0.26)	0.65
*F2RL3*_CpG2 (cg03636183)	-0.019 (-0.28, 0.24)	0.88	-0.098 (-0.40, 0.21)	0.50
*AHRR*_CpG1	0.053 (-0.47, 0.57)	0.83	-0.096 (-0.70, 0.50)	0.73
*AHRR*_CpG2	0.057 (-0.45, 0.57)	0.82	-0.093 (-0.67, 0.48)	0.73
*AHRR*_CpG3 (cg05575921)	0.05 (-0.53, 0.63)	0.86	-0.047 (-0.61, 0.51)	0.86
**∑2-,3-OH-Phe (µg/g crea.)**				
Relative telomere length	0.00060 (-0.0022, 0.0034)	0.65	-0.000074 (-0.0029, 0.0028)	0.96
Relative mtDNAcn	0.000048 (-0.0061, 0.0062)	0.99	0.0013 (-0.0051, 0.0077)	0.67
*F2RL3*_CpG1	-0.051 (-0.24, 0.14)	0.58	-0.051 (-0.24, 0.14)	0.57
*F2RL3*_CpG2 (cg03636183)	-0.033 (-0.21, 0.15)	0.70	-0.048 (-0.23, 0.13)	0.57
*AHRR*_CpG1	0.14 (-0.21, 0.50)	0.41	0.091 (-0.25, 0.43)	0.58
*AHRR*_CpG2	0.11 (-0.25, 0.46)	0.53	0.051 (-0.28, 0.38)	0.75
*AHRR*_CpG3 (cg05575921)	0.11 (-0.29, 0.52)	0.56	0.085 (-0.23, 0.40)	0.58
**4-OH-Phe (ng/g crea.)**				
Relative telomere length	0.0000070 (-0.000014, 0.000028)	0.50	-0.00000035 (-0.000024, 0.000023)	0.98
Relative mtDNAcn	0.000013 (-0.000033, 0.000059)	0.56	0.000024 (-0.000024, 0.000072)	0.30
*F2RL3*_CpG1	0.00056 (-0.00088, 0.0020)	0.42	-0.00014 (-0.0017, 0.0014)	0.85
*F2RL3*_CpG2 (cg03636183)	0.00072 (-0.00060, 0.0020)	0.27	0.000030 (-0.0015, 0.0015)	0.97
*AHRR*_CpG1	0.0018 (-0.00084, 0.0044)	0.17	0.00076 (-0.0021, 0.0036)	0.57
*AHRR*_CpG2	0.0017 (-0.00090, 0.0043)	0.19	0.00068 (-0.0021, 0.0034)	0.60
*AHRR*_CpG3 (cg05575921)	0.0021 (-0.00078, 0.0050)	0.14	0.00081 (-0.0018, 0.0035)	0.52
**∑OH-Phe (µg/g crea.)**				
Relative telomere length	0.00035 (-0.0013, 0.002)	0.66	-0.000037 (-0.0018, 0.0018)	0.97
Relative mtDNAcn	0.00056 (-0.0031, 0.0042)	0.75	0.00091 (-0.0030, 0.0049)	0.62
*F2RL3*_CpG1	-0.021 (-0.14, 0.094)	0.70	-0.031 (-0.15, 0.089)	0.59
*F2RL3*_CpG2 (cg03636183)	-0.011 (-0.12, 0.096)	0.83	-0.032 (-0.14, 0.080)	0.54
*AHRR*_CpG1	0.071 (-0.14, 0.29)	0.49	0.028 (-0.19, 0.25)	0.79
*AHRR*_CpG2	0.058 (-0.15, 0.27)	0.57	0.012 (-0.20, 0.22)	0.90
*AHRR*_CpG3 (cg05575921)	0.062 (-0.18, 0.30)	0.59	0.033 (-0.17, 0.24)	0.74

Abbreviations: mtDNAcn (mitochondrial DNA copy number); ∑2-,3-OH-Phe (sum of 2- and 3-hydroxyphenanthrene); ∑OH-Phe (Sum of 1-, 2-, 3-, and 4-hydroxyphenanthrene)
*There was 1 missing case for some of the outcomes (e.g. *AHRR*_CpG3_cg05575921)

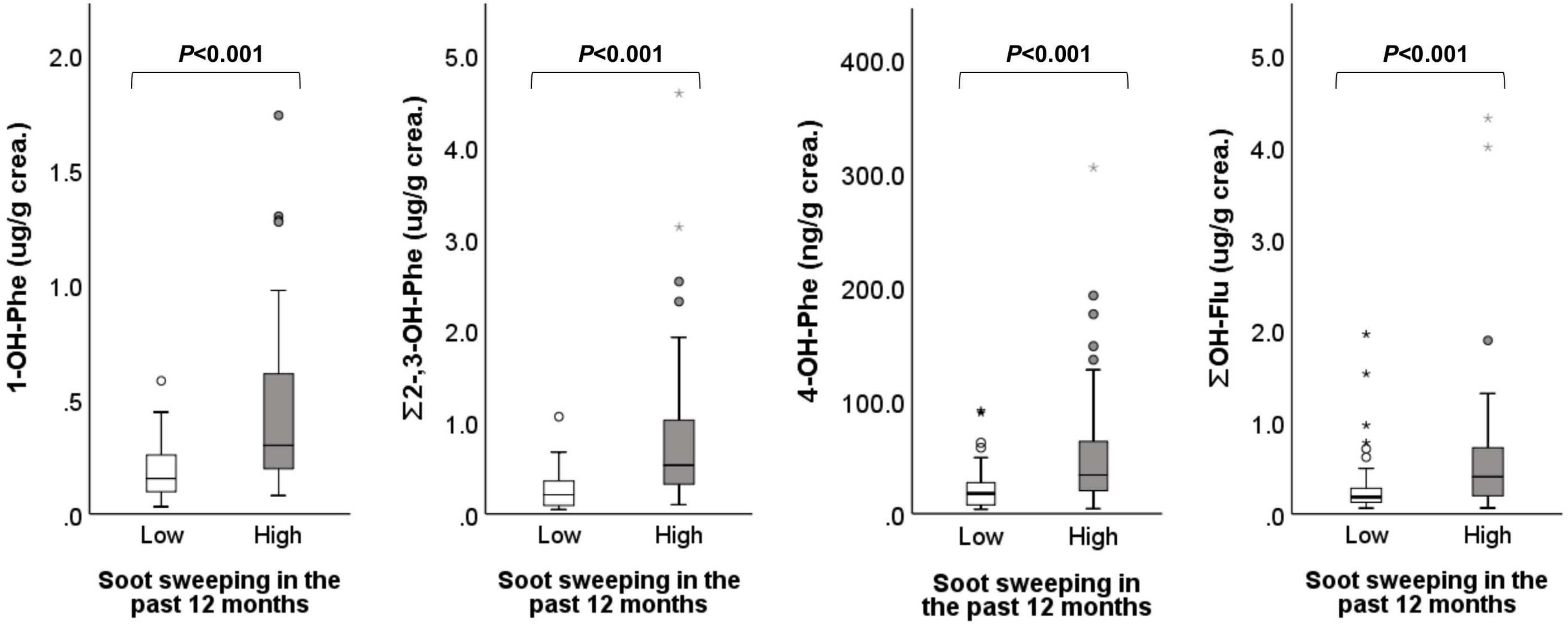

Fig. S1. Differences in urinary PAH metabolite concentrations for chimney sweeps who spent ≥50% of their time doing soot sweeping in the past 12 months versus those who spent <50% (excluding current smokers). *P*-value was derived from Mann-Whitney U test. ∑2-,3-OH-Phe refers to the sum of 2- and 3-hydroxyphenanthrene and similarly, ∑OH-Flu refers to the sum of 2- and 3-hydroxyfluorene.

Alhamdow A, *et al. Occup Environ Med* 2020; 77:488–495. doi: 10.1136/oemed-2020-106413

References

Alhamdow A, Lindh C, Hagberg J et al. DNA methylation of the cancer-related genes *F2RL3* and *AHRR* is associated with occupational exposure to polycyclic aromatic hydrocarbons. *Carcinogenesis* 2018;39:869-878.
